# Innovation and application of Large Language Models (LLMs) in dentistry – a scoping review

**DOI:** 10.1038/s41405-024-00277-6

**Published:** 2024-12-01

**Authors:** Fahad Umer, Itrat Batool, Nighat Naved

**Affiliations:** 1 Associate Professor, Operative Dentistry & Endodontics, Aga Khan University Hospital, Karachi, Pakistan; 2 Resident, Operative Dentistry & Endodontics, Aga Khan University Hospital, Karachi, Pakistan

**Keywords:** Dentistry, Occupational health

## Abstract

**Objective:**

Large Language Models (LLMs) have revolutionized healthcare, yet their integration in dentistry remains underexplored. Therefore, this scoping review aims to systematically evaluate current literature on LLMs in dentistry.

**Data sources:**

The search covered PubMed, Scopus, IEEE Xplore, and Google Scholar, with studies selected based on predefined criteria. Data were extracted to identify applications, evaluation metrics, prompting strategies, and deployment levels of LLMs in dental practice.

**Results:**

From 4079 records, 17 studies met the inclusion criteria. ChatGPT was the predominant model, mainly used for post-operative patient queries. Likert scale was the most reported evaluation metric, and only two studies employed advanced prompting strategies. Most studies were at level 3 of deployment, indicating practical application but requiring refinement.

**Conclusion:**

LLMs showed extensive applicability in dental specialties; however, reliance on ChatGPT necessitates diversified assessments across multiple LLMs. Standardizing reporting practices and employing advanced prompting techniques are crucial for transparency and reproducibility, necessitating continuous efforts to optimize LLM utility and address existing challenges.

## Introduction

Generative Artificial Intelligence (AI) represents a groundbreaking advancement in machine learning, particularly through the development of Large Language Models (LLMs) [1]. These sophisticated systems are designed to generate human-like text by leveraging vast datasets and complex algorithms [2]. LLMs, utilize transformer architectures to process and predict text, enabling them to perform a wide range of tasks from text completion to translation and summarization [3]. These models operate by segmenting input data into tokens and using self-attention mechanisms to understand and generate coherent sequences of text, thereby mimicking human-like understanding and communication [4].

LLMs employing deep learning algorithms process and comprehend natural language, enabling pattern recognition, translation, or generation of text and diverse content [5]. They have revolutionized healthcare by enhancing the efficiency, accuracy, and accessibility of medical services [6, 7]. Their ability to process and analyze large volumes of clinical data, understand complex medical terminologies, and generate detailed medical reports has significantly improved clinical documentation and patient care.

LLMs which have rapidly advanced the general field of healthcare, are also poised to make significant contributions within dentistry—an area that has only begun to explore their potential. For instance, it can automate the generation of medical records and progress notes, streamlining administrative tasks for dental practitioners [8]. Additionally, it can assist in summarizing complex research papers, extracting key information to keep clinicians updated on the latest developments [9]. Moreover, LLMs are increasingly being utilized in patient query handling, with the development of chatbots and virtual assistants that can provide accurate and timely responses to patient inquiries [10]. This kind of support aligns with dentistry’s high patient-interaction environment, where timely and accurate information is essential for patient satisfaction and adherence to care protocols [11]. Through these applications, LLMs not only augment the capabilities of dental professionals but also contribute to more informed decision-making and better patient outcomes.

To enhance the performance of LLMs in domain-specific tasks compared to general-purpose models, various prompting strategies can be employed [12]. Advanced prompting techniques such as role prompting, one-shot, few-shot, or chain-of-thought prompting provide context-rich inputs that guide the model to generate more relevant and precise responses [13]. Embedding techniques, which represent words or phrases in vector space, facilitate the model’s understanding of context and relationships between terms, improving its ability to handle specialized medical vocabulary [14]. Retrieval-Augmented Generation (RAG) combines LLMs with external knowledge sources, retrieving relevant information to support the generation process, thereby increasing the reliability and specificity of the outputs [15]. By integrating these strategies, LLMs can overcome the limitations of general-purpose models, delivering more accurate and contextually appropriate responses in specialized fields such as dentistry. The operational definitions of key prompting strategies and frequently used terminologies in LLMs are presented in Supplementary Table 2.

The deployment of Large Language Models (LLMs) in healthcare and dentistry can be understood through different levels, reflecting stages of integration and maturity, as described by Zhang et al. in their study on the development maturity of clinical artificial intelligence research [16]. At Level 1, LLMs are in the experimental phase, primarily focused on algorithm development and initial testing. Level 2 involves early adoption, where models are tested in controlled environments to validate their efficacy and reliability. By Level 3, LLMs are integrated into practical applications, often referred to as the “model into device” stage, where they begin to interact with real-world data and users. Level 4 represents mature deployment, where LLMs are fully embedded within healthcare systems, continually monitored, and refined to ensure optimal performance and reliability in diverse clinical settings.

By employing these advanced techniques and progressing through the stages of deployment, LLMs hold the potential to significantly advance healthcare, offering tailored solutions that address the unique challenges and requirements of the medical field. While there has been considerable research on the applicability of LLMs in various medical domains, their integration within dentistry remains underexplored [17–19]. Therefore, this scoping review aims to systematically evaluate the current literature on the application of LLMs in dentistry. By synthesizing the existing evidence, this review seeks to elucidate the diverse use cases, identify research gaps, and assess the methodologies employed such as evaluation metrics used in studies utilizing LLMs within dental practice. Furthermore, the review will examine the type of LLM model used (general purpose models versus prompting strategies employed) as well as offering insight into the current state of LLM integration in dental practice. Through a meticulous review, we aim to advance knowledge in this field and guide the effective integration of LLMs into dental practice for optimal outcomes.

## Materials and methods

The scoping review was carried out following the established standards and guidelines outlined in the Preferred Reporting Items for Systematic Reviews and Meta-Analysis with the associated extension for Scoping Reviews (PRISMA-SCr). The protocol can be accessed through the Open Science Framework platform (https://osf.io/vqjz3).

### Search strategy

The authors, in collaboration with a medical information specialist from Aga Khan University Hospital, Pakistan, developed a comprehensive search strategy utilizing various combinations of key search terms. A pilot search was conducted by the authors to refine the search strategy. Initially, the search produced a broad range of studies, many of which were tangentially related to the main topic. Additionally, the pilot search indicated that certain databases yielded more focused results; for example, IEEE Xplore provided highly relevant technical papers, while PubMed included a mix of broader dental applications. Adjusting the inclusion criteria to emphasize empirical studies related to dental practice rather than theoretical discussions further narrowed the results, ensuring that the final search strategy was both comprehensive and directly aligned with the research objectives.

### Literature search

An extensive literature search was conducted in March 2024 through three electronic databases: PubMed (NLM), Scopus, and Institute of Electrical and Electronics Engineers (IEEE) Xplore. Additionally, a manual search was performed on Google Scholar to identify any additional literature addressing the review questions.

### Search terms

The following search terms were used to identify the relevant literature:

Large Language Models OR LLM OR LLMA 2 OR ChatGPT OR Generative Artificial Intelligence OR Generative AI OR Chatbots OR Natural Language Processing OR NLP OR Google Bard OR PaLM OR PaLM 2 OR Gemini AND dental OR dentistry OR restorative dentistry OR endodontics OR prosthodontics OR periodontics OR maxillofacial surgery OR oral surgery OR orthodontics

### Screening process

Article citations were exported to the Endnote reference manager version 20.0 (Clarivate Analytics) where duplicate references were removed. Two authors (IB and NN) screened the titles, abstracts, and full texts of the studies according to the predetermined inclusion criteria. Any disagreement between the two was resolved through discussion with the third author (FU). The data were added to a calibrated proforma independently by all three authors. Additionally, the extracted information was rechecked by the senior author (FU).

### Review questions

1. What are the specific applications of Large Language Models (LLMs) in various dental specialties, and how have they been utilized to date?
2. What evaluation metrics are employed in studies assessing the performance of LLMs in dental practice?
3. What evidence exists regarding the accuracy and efficiency of LLMs in dentistry?
4. What type of LLM models were used in the studies, the general-purpose models or with advanced prompting strategies?
5. What is the current state of LLM integration (level of deployment) in dental practice?

### Data extraction

A customized proforma was designed by the authors to extract the following information from included studies:

1. Study details (title, authors, journal of publication, year of publication)
2. Study characteristics (specialty/field and application)
3. Type of LLM model/algorithm used (GPT, Bard, Llama, Bloom)
4. Evaluation metrics utilized in the individual studies
5. Prompting strategies or training used (fine-tuning, embedding, RAG)
6. LLM deployment level

### Inclusion criteria

- Primary studies utilizing LLMs in dental practice
- Studies in English language

### Exclusion criteria

- Reviews, editorials, commentaries, and conference proceedings
- Studies available as abstract only
- Studies registered as protocols

## Results

**Fig. 1:**
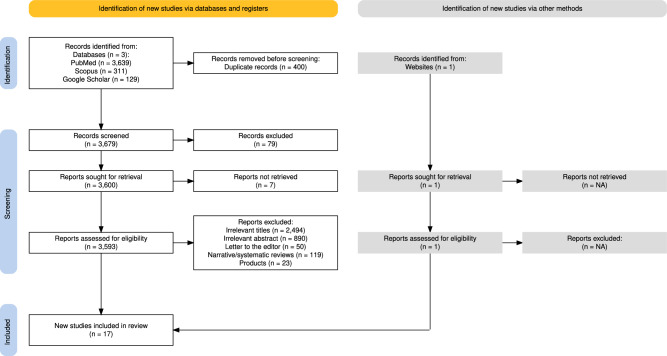
PRISMA flowchart. The figure illustrates the search and retrieval processes of studies via PubMed, Scopus, Google Scholar and IEEE Xplore. After comprehensive screening, 17 studies were found to be eligible and included in the analysis.

Following a detailed manual and electronic literature search, 4079 records were identified. After removal of 400 duplicates, the remaining 3679 records were screened for relevance and 79 articles were excluded. A total of 3593 articles underwent final screening for eligibility check and after excluding narrative/systematic reviews, letter to the editor, product reviews and papers with irrelevant titles and abstracts, 17 studies fulfilling the inclusion criteria were included in the analysis. The PRISMA flowchart for screening process is presented in Fig. 1.

The characteristics of included studies extracted on a customized proforma is presented in Supplementary Table 1.

ChatGPT was the predominant large language model (LLM) utilized in 15 studies [20–34]. In contrast, other less frequently utilized AI tools were Bing, Google Bard, Open Evidence, and Medi Search [24, 29]. The primary objective of most of the studies (15 studies) was to address post-operative patient queries [20–29, 31, 33–36]. Additionally, one study focused on generating radiology reports, and another aimed at diagnosing conditions based on patient history and radiographic findings [30, 32]. The specialty-wise distribution of the included studies revealed that majority were within the domains of Oral and Maxillofacial Surgery and Orthodontics [21, 24–26, 30, 31, 33]. This was followed by studies in other domains such as Endodontics, Periodontics, General Dentistry, Maxillofacial Radiology, Prosthodontics, Dental Public Health, and Dental Radiology [20, 22, 23, 27, 29, 30, 32, 34–36].

**Fig. 2:**
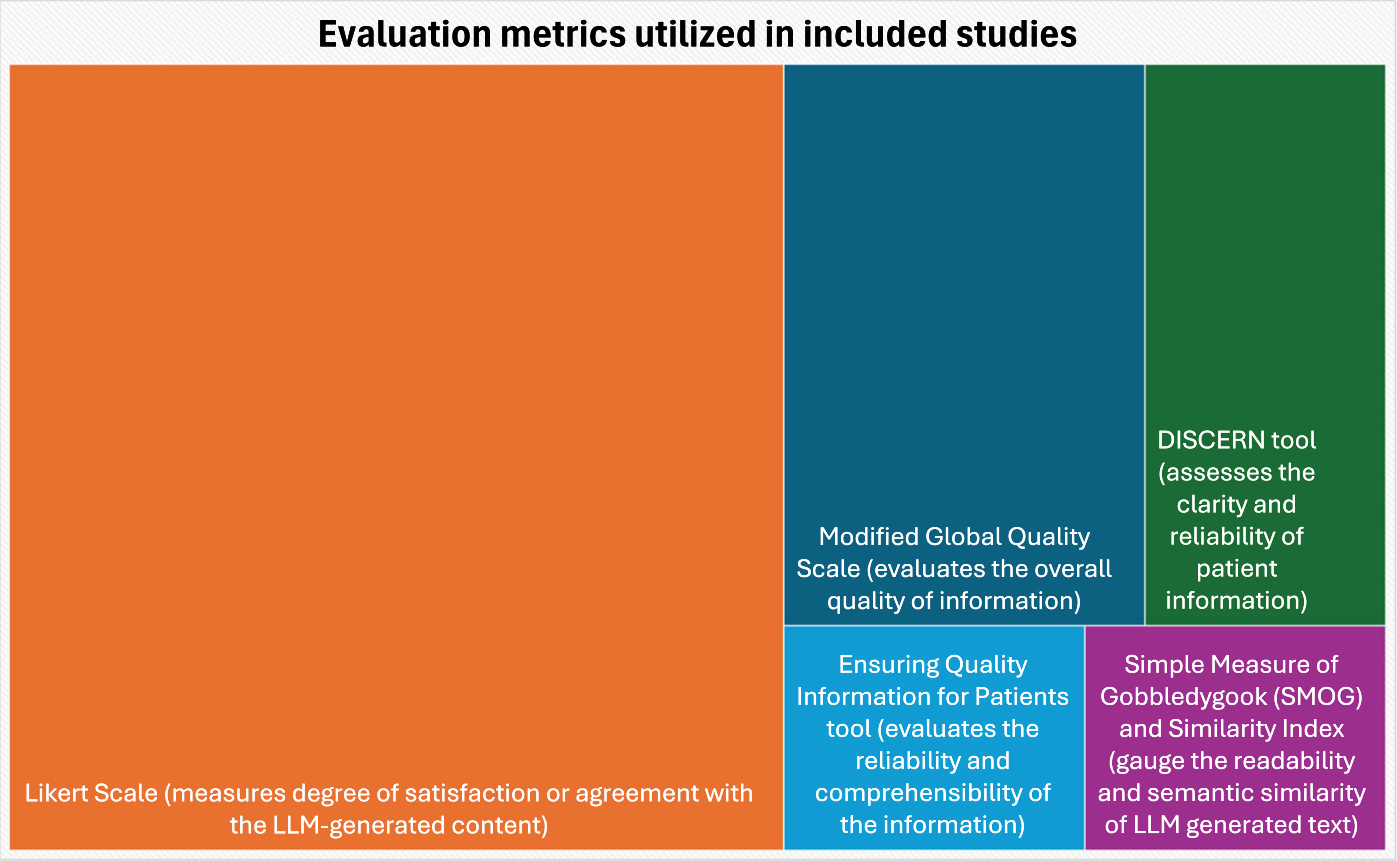
Evaluation metrics utilized in the included studies. The figure shows a brief description of the evaluation metrics used. The size of each colored box represents the number (weightage) of studies utilizing the individual metrics.

Various evaluation metrics were employed across the included studies on the use of LLMs in dentistry. These included Likert Scale (9 studies), the Modified Global Quality Scale (3 studies), DISCERN tool (2 studies), Ensuring Quality Information for Patients tool (1 study), and Simple Measure of Gobbledygook (SMOG) and Similarity Index (1 study). The commonly used evaluation metrics with a brief description of each are presented in Fig. 2.

**Fig. 3:**
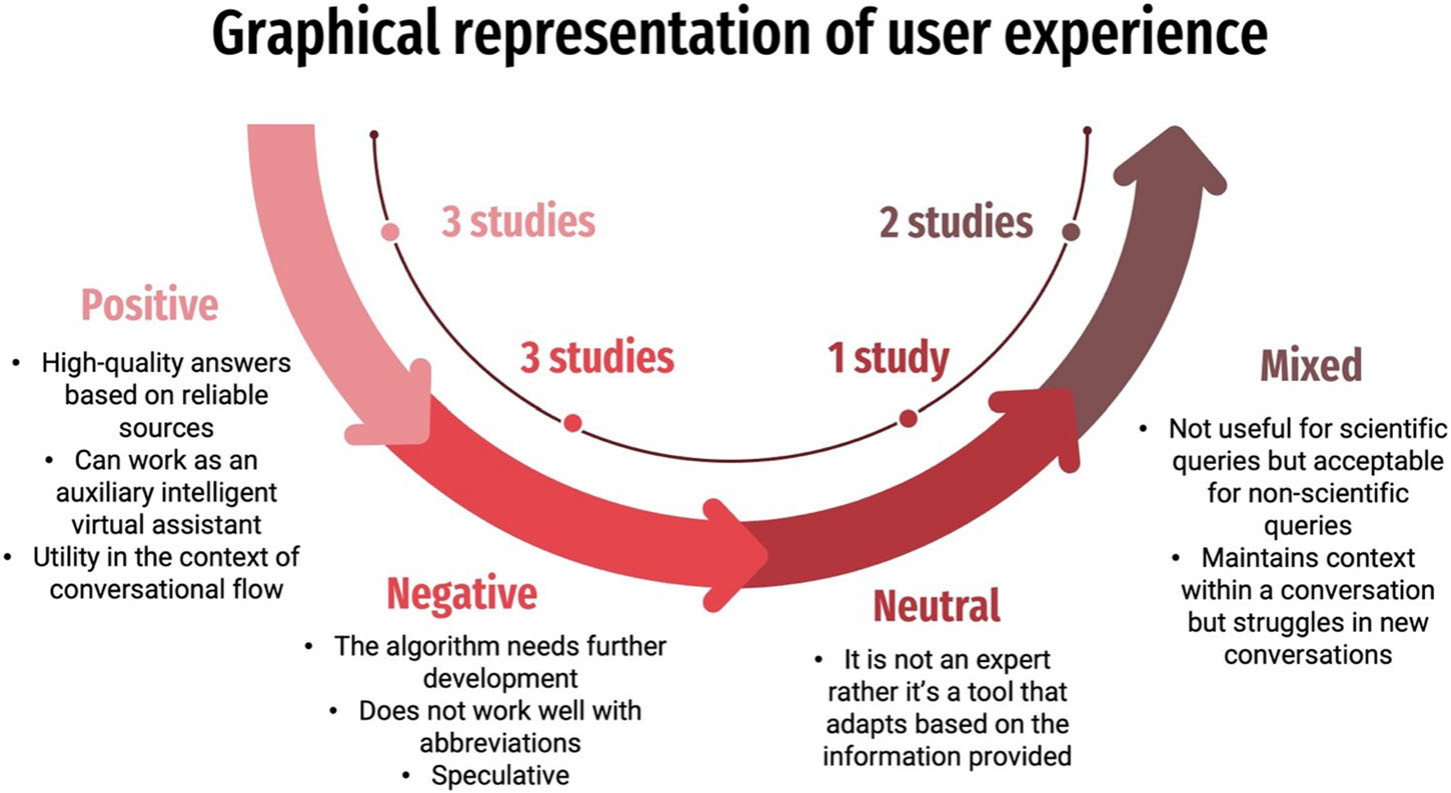
Graphical representation of user experience. The figure shows the positive, negative, neutral and mixed perspective of the human evaluators reported in the included studies.

Interestingly, only two studies employed advanced prompting strategies such as zero-shot and chain-of-thought prompting [28, 32]. No prompting strategy was used in the remaining studies. Regarding the level of maturity according to the stage of development depicting the deployment of these LLMs, it was found that nearly all studies were at level 3 of deployment (model into device stage). Moreover, the evaluators in almost all the included studies were human dental experts. Their user experiences (positive, negative, mixed, or neutral) as reported in the individual studies are presented in Fig. 3.

## Discussion

The justification for conducting a scoping review on the use of LLMs in dentistry derives from the need to thoroughly comprehend the current state of research in this field, identify any gaps or limitations and offer suggestions for future research. A scoping review is particularly beneficial and preferred over a systematic review when there are open study questions and no predefined PICOs, as this allows researchers to broadly explore a topic, identify and clarify key terms, and visualize the landscape of research.

### Application of LLMs across dental specialties

Through the course of our scoping review, we found extensive utilization of LLMs in domains such as Dental Public Health, Oral/Maxillofacial Surgery, Periodontology, Orthodontics, General Dentistry, Oral Surgery, Endodontics, Dental Radiology, Preventive Dentistry, and Prosthodontics. However, it is noteworthy that certain domains, such as Pediatric Dentistry, Implant Dentistry, and Oral Pathology, have not been extensively documented in the literature regarding their use of LLMs up to the time of conducting this scoping review. Moreover, while the studies focused on post-operative patient queries, generating radiology reports, and diagnosis based on patient history and radiographic findings, several critical aspects of dental healthcare were not covered. These include treatment planning, patient education, emergency dental care, integration with electronic health records (EHRs), and telehealth applications. Exploring the potential of LLMs in these areas could further enhance patient care, improve treatment outcomes, and increase efficiency in clinical practices.

### Predominantly employed LLM in dental practice

In our scoping review, ChatGPT emerged as the predominant Large Language Model (LLM) utilized in various studies, as opposed to other available models like Llama 2, Gemini, Claude 2, Mixtral 8x7B, and Falcon. This popularity could be attributed to ChatGPT’s user-friendly interface, 24/7 accessibility, and the advantage of being the first LLM to enter the market [37]. While ChatGPT’s extensive usage offers advantages, it is important to recognize potential limitations in solely relying on it as this may overlook the unique features and potential advantages offered by other LLMs.

It is notable that authors sometimes omitted specifying the version of ChatGPT employed in their research. This omission could potentially pose a challenge in replicating and comparing study findings, as different versions of ChatGPT may exhibit varying performance characteristics [38]. To ensure transparency and reproducibility in LLM research, it is recommended that authors explicitly mention the versions of all LLM models utilized in their studies.

### Challenges associated with general-purpose models

We observed that most studies utilized general-purpose models of ChatGPT, which are trained on a wide corpus of internet text. While these models performed well on general question-and-answer tasks, they often struggled with domain-specific technical questions. This limitation underscores the importance of employing advanced prompting techniques to enhance the performance of LLMs in specialized domains. Techniques such as role prompting, which involves adding a system message or utilizing different prompting strategies like one-shot, few-shot, or multi-shot prompts, can provide richer context and improve model understanding [13]. However, in our review, we found that only two studies incorporated advanced prompting techniques, highlighting a potential area for further exploration and development in LLM research within dentistry [28, 32].

### Concerns regarding reliability of generated information

The studies reviewed in our paper indicate that the information generated by LLMs lacked references, raising concerns about its reliability. This issue can be addressed by employing retrieval-augmented generation techniques (RAG), which integrate retrieved knowledge with the model’s generation process [15]. Interestingly, none of the included studies in our review utilized any LLM modification techniques, such as fine-tuning or RAG, suggesting a potential avenue for future research to enhance the trustworthiness and accuracy of LLM-generated information in dentistry.

### Maturity level of LLM deployment

The evaluation of the level of maturity in the deployment of LLMs in dental practice revealed that nearly all studies were at level 3 of deployment, which corresponds to the “model into device” stage. This stage indicates that the LLMs have moved beyond theoretical or pilot phases (levels 1 and 2, which involve initial development and early testing) and are being integrated into practical, usable applications within the healthcare setting. It demonstrates that the models have undergone sufficient development and validation to be trusted in real-world scenarios. However, achieving level 3 also highlights the need for continuous monitoring and refinement to ensure the models maintain accuracy, reliability, and relevance as they interact with actual users and encounter diverse real-world data (level 4) [16]. While most studies focused on assessing the output of LLMs against expert knowledge, there is untapped potential for further research to explore the utility of these models in real-world deployment among patients and healthcare providers. Understanding user acceptability and the practical application of LLMs beyond controlled research settings is crucial for informing their integration into clinical practice.

### Lack of standardization of assessment tools

A notable shortcoming was that assessments in the included studies were conducted by subject-level experts using customized assessment tools tailored for each study, including Likert scales, modified Discern instruments, or modified Global Quality Scores (GQS). This lack of standardization precludes the homogenization of results across studies, making it challenging to compare findings effectively. Therefore, there is a pressing need for the development of a standardized assessment tool to facilitate better comparison of results and enhance the validity and reliability of evaluations across different studies. Furthermore, employing quantitative scales rather than Likert scales could provide a more objective means of quantifying outputs; however, it is important to acknowledge that quantitative measures also come with their own limitations, such as potential oversimplification of complex constructs and challenges in accurately capturing subtle variations in responses.

### Need for standardized reporting

We observed a lack of standardized terminologies for assessment in the studies reviewed. Terms like accuracy, reliability, content analysis, validity, among others, were employed without clear definitions or consistent usage. This variability could potentially lead to confusion and hinder comparability across studies. It is essential for the research community and individual researchers to explicitly define these terms within the context of their studies, ensuring consistency and clarity in reporting. By adhering to accepted terminologies and valid performance metrics in a standardized manner, researchers can enhance the reliability and comprehensibility of their findings.

This is the first review of its kind that methodically explores the trends and progress of LLM related research in dental practice. However, the inclusion of only three databases in the search may have resulted in the omission of some relevant articles. Additionally, to ensure a wider inclusion of studies, the research questions posed in the review were intentionally broad. Lastly, while findings were extracted following a predefined methodology, some were added in an ad hoc manner to enhance the overall yield of our review.

## Conclusion

Large Language Models have the potential to transform healthcare and dentistry by enhancing patient care and improving administrative efficiency. This includes providing accurate patient query responses, diagnostic assistance, and streamlining documentation processes. While ChatGPT was the frequently employed tool, diversifying assessments across various LLMs is essential for a comprehensive understanding of their capabilities. Moreover, to optimize the utility of LLMs, future research should focus on specific applications in dentistry and developing guidelines for effective integration. Furthermore, addressing challenges such as privacy, ethical use of the data, and training of practitioners will enable the dental profession to maximize the benefits of LLMs in clinical practice.

## Supplementary information

Supplementary Tables

PRISMA Checklist

## Data Availability

The data analyzed during the current study are presented in Supplementary Table 1.
