# Supplementary material for: Innovation and application of Large Language Models (LLMs) in dentistry – a scoping review
Source: BDJ Open. 2024 Dec 1;10:90. doi: 10.1038/s41405-024-00277-6 (PMC11609263; doi:10.1038/s41405-024-00277-6)
Supplement: Supplementary file 1 — Supplementary Tables [file 41405_2024_277_MOESM1_ESM.pdf]

SI Table 1: Characteristics of included studies

S. No.	Author (year)	Journal (impact factor)	Specialty (field)	Type of LLM	Level of deployment	Objective	Evaluation metrics	Prompting strategies	LLM modifying technique	System message	Evaluator	Conclusion	Limitations	User experience
1.	Mehmet et al (2023) (1)	FLUORIDE-QUARTERLY REPORTS	Dental public health	Chat GPT (version not specified)	3	Aims to compare the content and information level of answers provided by ChatGPT to frequently asked questions about fluoride, as determined by the American Dental Association (ADA), with the answers given by the ADA,	None	None	None	None	Not disclosed	The accuracy and reliability of the answers given by the applications developed with artificial intelligence (AI) are of great importance, and it has been seen that the answers given by ChatGPT to the questions asked about fluoride are sufficient	None specified	None shared

						qualitatively.						and reliable.		
2.	Yunus Balel (2023) (2)	J Stomatol Oral Maxillofac Surg	Oral/ Maxillofacial Surgery	Chat GPT (version not specified)	3	The aim of this study is to assess the usability of the information generated by chatGPT in oral and maxillofacial surgery.	Human (Modified Global Quality Scale)	None	None	None	Human Experts surgeons	In conclusion, ChatGPT has significant potential as a tool for patient information in oral and maxillofacial surgery.	None specified	The surgeons who participated in our study were cautious about using ChatGPT in oral and maxillofacial surgery, and many of them felt that this algorithm needed further development.
3.	Osman et al (2023) (3)	Cureus	Periodontology	Chat GPT (version not specified)	3	The aim of this study is to evaluate the accuracy and completeness of	Likert scale 1-6 accuracy of response ; completeness of response	None	None	None	Humans, 20 periodontist	Even though ChatGPT cannot provide 100% accurate and comprehensive	One of the limitations of our study is the small number of periodontology profession	None shared

						the answers given by Chat Generative Pre-trained Transformer (ChatGPT) (OpenAI OpCo, LLC, San Francisco, CA), to the most frequently asked questions on different topics in the field of periodontology.	likert scale 1-3					findings without expert oversight, it is evident that patients in the field of periodontology can still use it for informational purposes by accepting some error risks.	als who assessed the responses. Furthermore, the scope of our study was limited to the examination of queries related exclusively to the field of periodontology in dentistry, which restricts the overall applicability of our evaluation to ChatGPT.	
4.	Raif et al (2023) (4)	Cureus	Periodontology	GPT -4	3	The aim of this study is to evaluate the responses returned by	DISCERN instrument	None	None	None	Human Experts surgeons 1 periodontist, 2 general dentist	The responses generated by ChatGPT-4 to patients' information	The study focused on topics with a high search volume related to Periodontal disease. However,	None shared

						ChatGPT-4, an AI chatbot, to queries related to PD based on Google Trends data in the last year						requests were 'good' in terms of quality and could be considered satisfactory. Although ChatGPT-4 provided incomplete or insufficient information about the 'treatment choices' section of the DISCERN instrument,	PD, which is a multifactorial disease, has different types. The ability of ChatGPT-4 to provide information about these different diseases/situations could not be evaluated.	
5.	Sarah et al (2024) (5)	Angle Orthod.	Orthodontics	Chat GPT (version not specified)	3	To assess the accuracy of ChatGPT answers concerning orthodontic clear aligners.	Modified four-point scale as follows: 1: Objectively true; 2: Selected facts; 3:	None	None	None	Human expert Orthodontist 5	Attempts should be made to improve the robustness of these AI models prior to their integration in the	Validation of ChatGPT may not necessarily apply to other AI models.	ChatGPT used in this research was not a useful tool for generating answers to scientifi

							Minimal Facts; 4: False					healthcare profession.		c queries. On the other hand, acceptable accuracy levels were observed for answers to questions concerning knowledge, satisfaction, compliance, and cost-effectiveness.
6.	Ebru et al (2023) (6)	J Stomatol Oral Maxillofac Surg	Oral and Maxillofacial surgery	ChatGPT-4, OpenEvidence, MediSearch	3	The aim of the current study is to evaluate the quality, reliability, readability, and	Ensuring Quality Information for Patients (EQIP) tool, Reliability Scoring System	None	None	None	Not mentioned	AI-based chatbots with a variety of features have usually provided answers with high quality, reliability,	Language restrictions to English. Data validity affected by chatbot updates. Potential hallucinations in	OpenEvidence and MediSearch, specifically developed for the fields of health

						similarity of data provided by different AI-based chatbots in the field of orthognathic surgery	(adapted from DISCERN), Global Quality Scale (GQS), Simple Measure of Gobbledygook (SMOG) and Similarity Index					and difficult readability to questions that patients may pose in the field of orthognathic surgery	ChatGPT responses. Performance variability among chatbot models. Limitations in providing creative or human-like responses	and biology, provide appropriate answers by relying on articles from the literature.
7.	Douglas (2022) (7)	IEEE Xplore	General dentistry	Chatbot (WhatsApp messaging application)	3	Chatbot use for pre-triage procedures: a case study at a free-service university dental clinic	the Post-Study System Usability Questionnaire (PSSUQ) evaluated using Likert scale	None	None	None	15 dental clinic users	92% of the values assigned in the PSSUQ were greater than 6, demonstrating good performance and user satisfaction. In addition, the average PSSUQ values were 6.66, being 6.74 for	Not mentioned	Not mentioned

												System Utility, 6.56 for Information Quality and 6.68 for Interface Quality. Which demonstrated that the chatbot was able to instruct users during pre-triage in a simple and easy way.		
8.	Ana Suarez et al (2024) (8)	Computational and Structural Biotechnology Journal	Oral Surgery	ChatGPT-4	3	This study aimed to assess whether ChatGPT-4 could provide accurate and reliable answers to general dentists in the field of	Three-point Likert scale	Yes	Prompting	Imagine that you are an oral surgeon and I am a general dentist. Please answer the following question accurately and directly, without rambling	Two postgraduate dentists specialized in oral surgery	ChatGPT in its current state should not be used indiscriminately	ChatGPT, by its nature, does not specify the sources of its information and cannot access recently updated documents. A validated scale was	Proper training from validated sources and monitoring by expert oral surgeons, ChatGPT has the

						oral surgery, and thus explore its potential as an intelligent virtual assistant in clinical decision making in oral surgery.				or creative answers			not used in this study. This limitation should be taken into account when evaluating the conclusions and practical applications derived from this study.	potential to become an auxiliary intelligent virtual assistant
9.	Ana Suárez (2023) (9)	International Endodontic journal	Endodontics	ChatGPT-4	3	The aim of this study was to evaluate the consistency and accuracy of ChatGPT-generated answers to clinical questions in endodontics,	Proportion, Chi square test, Confidence interval	None	None	None	Human experts	Currently, ChatGPT is not capable of replacing dentists in clinical decision-making. As ChatGPT's performance improves through deep learning, it is expected to become more useful and	ChatGPT is a language model designed for a general audience and was not specifically trained for the field of endodontics	None

						compared to answers provided by human experts						effective in the field of endodontics		
10.	Maximilian et al (2024) (10)	Dentomaxillofacial radiology	Dental Radiology	Content-aware chatbot based on GPT-3.5-Turbo and GPT-4	3	To develop a content-aware chatbot based on GPT-3.5-Turbo and GPT-4 with specialized knowledge on the German S2 Cone-Beam CT (CBCT) dental imaging guideline and to compare the performance against humans.	5-point Likert scale	Yes	Zero-shot learning, for this, German S2 guidelines for CBCT was utilized via vectorized embeddings and established automatic content retrieval.	QA_PROMPT (We have provided contextual information. Given this information, please answer the following question: question text Task: answer the question based on consensus-based recommendations. Explain the answer!	Four practitioners in dental imaging, two early career and two experienced	A content-aware chatbot based on GPT-4 was able to provide reliable recommendations according to the German S2 Cone-Beam CT dental imaging guideline at a level comparable to experienced practitioners.	This study is limited by focus on the German guidelines and thus the German language	None
11.	Samerchit et	JOURNAL OF		Wowbot - AI	3	Evaluate the	0-10 point for	None	None	None	Dental experts	Chatbots can be	First, both	Useful

	al (2022) (11)	MEDICAL INTERNET RESEARCH	Preventive dentistry	chatbot behavior change model		effectiveness and usability of the chatbots before and during the COVID-19 pandemic.	satisfaction on scale					useful in toothbrush training.	studies used a pre-post design that may have a maturity bias; therefore, the chatbot's effectiveness in improving oral health behavior may be overestimated. Second, although our study was conducted with similar research methodology, the interview procedure and follow-up period differed.	for planning the overall conversational flow and creating more humanized chatbots.
12.	Hossein et al (2023) (12)	International Endodontic Journal	Endodontics	GPT 3.5, google bard, Bing	3	This study aimed to evaluate and compare	A modified Global Quality Score (GQS)	None	None	None	2 Endodontist	GPT-3.5 provided more credible	Not mentioned	Not mentioned

						the validity and reliabilit y of re-sponses provided by GPT-3.5, Google Bard, and Bing to frequentl y asked questions (FAQs) in the field of endodont ics.	Likert scale 5-1 higher score is better context and content was used for validity. The question s were repeated 3 times to check for reliabilit y (consiste ncy)					informatio n on topics related to endodonti cs compared to Google Bard and Bing.		
1 3.	Jyoti et al (2023) (13)	Cureus	Maxillof acial Radiolog y	GPT 3	3	GPT3 for radiology report writing	4 points Likert Scale and SWOT analysis	None	None	None	The author specialty not mention ed	This technolog y is a good and handy adjunct to the oral and maxillofac ial radiologist and a great tool in educating and creating awareness	The limitation of the study includes that this is a small study that queried only anatomical landmarks and features of pathologie s and their radiograph ic analysis	this LLM did not work well with abbrevia tions

												among the public/the community about the disease process.	by a single evaluator.	
14.	Delal et al (2023) (14)	AJO-DO	Orthodontics	Chat Gpt (version not specified)	3	This study aimed to evaluate the reliability and readability of ChatGPT's responses to orthodontics-related questions and the evolution of these responses in an updated version.	DISCERN tool	None	None	None	Two orthodontists	the reliability of the answers was found to be moderate according to the	Our study had some limitations. ChatGPT does not give the same answers, even for consecutive queries.	ChatGPT is able to maintain the "chat" in context. For example, if the question "What is Phase I and Phase II therapy?" is asked during a conversation about orthodon-tics, AI answers the question in the context of

														orthodo n- tics. Howeve r, it gives an answer in a totally different context when this question is asked in a complet ely new conversa tion.
1 5.	Yanni et al (2024) (15)	BMC oral health	Maxillof acial Radiolog y	Chat Gpt (version not specified)	3	This study aimed to assess the performa nce of OpenAI' s ChatGPT in generatin g diagnosis based on chief complain t and	Based on five-point Likert scale.	Yes	Chain of though t prompt ing	Yes	Two radiologi sts for benchma rking (ground truth) one radiologi st for evaluatio n	ChatGPT showed potential in generating radiograp hic diagnosis based on chief complaint and radiologic findings. However, the performan ce of	A restricted dataset that didn't fully capture the diversity of dental and maxillofac ial diseases.	Further more, ChatGP T tends to follow instructi ons rather than engage in genuine interacti on [24]. For instance, when

						cone beam computed tomography (CBCT) radiologic findings.						ChatGPT varied with task complexity, necessitating professional oversight due to a certain error rate.		the radiologic findings are insufficient, ChatGPT may make assumptions that can-not be derived from the radiologists' descriptions.
16.	Arjeta et al (2024) (16)	Journal of clinical medicine	Orthodontics	Chat Gpt (version not specified)	3	This study aims to investigate the accuracy and completeness of ChatGPT in answering questions and solving clinical scenarios of intercepti	Accuracy (1-6) precsion (1-3) likert scale	None	None	None	10 orthodontist and 10 PG students	The results showed a high level of accuracy and completeness in AI responses and a great ability to solve difficult clinical cases, but the answers were not	Only 10 orthodontist and 10 students were used to formulate the questions	ChatGPT is not a professor or an expert that independently understands the nuances of orthodontics; it is a tool that adapts its response

						ve orthodontics.						100% accurate and complete.		s based on the information and context provided by the user.
17.	Yolanda et al (2024) (17)	The Journal of prosthetic dentistry	Prosthodontics	chat GPT 4	3	The purpose of this study was to determine the performance of ChatGPT in generating answers about removable dental prostheses (RDPs) and tooth-supported fixed dental prostheses (FDPs).	using a 3-point Likert scale	None	None	None	2 prosthodontists	The results show that currently ChatGPT has limited ability to generate answers related to RDPs and tooth-supported FDPs. Therefore, ChatGPT cannot replace a dentist, and, if professionals were to use it, they should be aware of its limitations.	None	None

Table 1: Characteristics of included studies

References:

1. Buldur M, Sezer B. Can Artificial Intelligence Effectively Respond to Frequently Asked Questions About Fluoride Usage and Effects? A Qualitative Study on ChatGPT. FLUORIDE-QUARTERLY REPORTS. 2023;56(3).
2. Balel Y. Can ChatGPT be used in oral and maxillofacial surgery? J Stomatol Oral Maxillofac Surg. 2023;124(5):101471.
3. Babayiğit O, Tastan Eroglu Z, Ozkan Sen D, Ucan Yarkac F. Potential Use of ChatGPT for Patient Information in Periodontology: A Descriptive Pilot Study. Cureus. 2023;15(11):e48518.
4. Alan R, Alan BM. Utilizing ChatGPT-4 for Providing Information on Periodontal Disease to Patients: A DISCERN Quality Analysis. Cureus. 2023;15(9):e46213.
5. Abu Arqub S, Al-Moghrabi D, Allareddy V, Upadhyay M, Vaid N, Yadav S. Content analysis of AI-generated (ChatGPT) responses concerning orthodontic clear aligners. Angle Orthod. 2024;94(3):263-72.
6. Yurdakurban E, Topsakal KG, Duran GS. A comparative analysis of AI-based chatbots: Assessing data quality in orthognathic surgery related patient information. J Stomatol Oral Maxillofac Surg. 2023;125(5):101757.
7. Vidal DA, da Costa Pantoja LJ, de Albuquerque Jassé FF, Arantes DC, da Rocha Seruffo MC, editors. Chatbot use for pre-triage procedures: a case study at a free-service university dental clinic. 2022 IEEE Latin American Conference on Computational Intelligence (LA-CCI); 2022: IEEE.
8. Suárez A, Jiménez J, Llorente de Pedro M, Andreu-Vázquez C, Díaz-Flores García V, Gómez Sánchez M, et al. Beyond the Scalpel: Assessing ChatGPT's potential as an auxiliary intelligent virtual assistant in oral surgery. Comput Struct Biotechnol J. 2024;24:46-52.
9. Suárez A, Díaz-Flores García V, Algar J, Gómez Sánchez M, Llorente de Pedro M, Freire Y. Unveiling the ChatGPT phenomenon: Evaluating the consistency and accuracy of endodontic question answers. Int Endod J. 2024;57(1):108-13.
10. Russe MF, Rau A, Ermer MA, Rothweiler R, Wenger S, Klöble K, et al. A content-aware chatbot based on GPT 4 provides trustworthy recommendations for Cone-Beam CT guidelines in dental imaging. Dentomaxillofac Radiol. 2024;53(2):109-14.
11. Pithpornchaiyakul S, Naorungroj S, Pupong K, Hunsrisakhun J. Using a Chatbot as an Alternative Approach for In-Person Toothbrushing Training During the COVID-19 Pandemic: Comparative Study. J Med Internet Res. 2022;24(10):e39218.
12. Mohammad-Rahimi H, Ourang SA, Pourhoseingholi MA, Dianat O, Dummer PMH, Nosrat A. Validity and reliability of artificial intelligence chatbots as public sources of information on endodontics. Int Endod J. 2024;57(3):305-14.
13. Mago J, Sharma M. The Potential Usefulness of ChatGPT in Oral and Maxillofacial Radiology. Cureus. 2023;15(7):e42133.
14. Kılınç DD, Mansız D. Examination of the reliability and readability of Chatbot Generative Pretrained Transformer's (ChatGPT) responses to questions about orthodontics and the evolution of these responses in an updated version. Am J Orthod Dentofacial Orthop. 2024;165(5):546-55.
15. Hu Y, Hu Z, Liu W, Gao A, Wen S, Liu S, et al. Exploring the potential of ChatGPT as an adjunct for generating diagnosis based on chief complaint and cone beam CT radiologic findings. BMC Med Inform Decis Mak. 2024;24(1):55.
16. Hatia A, Doldo T, Parrini S, Chisci E, Cipriani L, Montagna L, et al. Accuracy and Completeness of ChatGPT-Generated Information on Interceptive Orthodontics: A Multicenter Collaborative Study. J Clin Med. 2024;13(3).
17. Freire Y, Santamaría Laorden A, Orejas Pérez J, Gómez Sánchez M, Díaz-Flores García V, Suárez A. ChatGPT performance in prosthodontics: Assessment of accuracy and repeatability in answer generation. J Prosthet Dent. 2024;131(4):659.e1-.e6.

Supplementary Table 2: Operational definition of various terminologies used in understanding LLMs.

Prompting	“Prompting" refers to the technique of providing specific input or instructions to guide the model's response generation process in the context of Large Language Models (LLMs). Prompting involves framing the input text in a way that elicits the desired output.
Zero-Shot Prompting	The model is given a task without any examples and is expected to generate the appropriate response based solely on the instructions in the prompt (N=0)
One-Shot Prompting	The model is provided with a single example of the task to guide its response (N=1)
Few-Shot Prompting	The model is provided with a few examples of the task along with the prompt (N≥2)<br><br>Research has shown that few-shot prompts outperform one-shot prompts, which outperform zero-shot prompts, and the authors use the term “in-context learning” to describe this phenomenon.
Chain-of-thought Prompting	This is similar to few-shot prompting but is structured in a way that encourages the model to think through the steps required to arrive at the answer, leading to more coherent and logical responses.
Fine tuning	Fine-tuning is the process of adjusting a pre-trained model on a specific, often narrower, dataset or task to enhance its performance in that particular domain.
RAG	Retrieval-Augmented Generation (RAG) is a technique used with Large Language Models (LLMs) that combines the capabilities of generative models with retrieval mechanisms to enhance the accuracy and relevance of the generated responses. This method involves retrieving relevant information from an external knowledge base and integrating it into the text generation process. RAG can provide more precise and contextually appropriate answers, especially in specialized domains where up-to-date and accurate information is crucial.

Hallucination	Hallucination in a model refers to the generation of content that strays from factual reality or includes fabricated information. Hallucination can occur when the model produces text that includes details, facts, or claims that are fictional, misleading, or entirely fabricated, rather than providing reliable and truthful information. This can be dangerous when LLMs are used in critical domains where accuracy and safety are important.
Prompt engineering	Prompt-engineering is the process of designing natural language specifications of a task, which are used to condition the LLM at inference time. The prompt format changes the model behavior and proposes particular formats
Misalignment	Alignment means that LLMs act in accordance with their human users' intentions. LLMs that are misaligned act differently from what their users want. This can also cause harm, such as giving wrong answers, generating biased outputs or discriminating results. Alignment involves tuning LLMs to encourage desired behaviors and discourage undesired ones.
Parameter efficient tuning	Parameter efficient tuning optimizes a small portion of the model parameters while keeping the rest fixed, drastically cutting down computation and storage costs. Fine-tuning the whole model is parameter inefficient as it always yields an entirely new model for each task.
